# A Rapid Fabrication Method of Large-Area MLAs with Variable Curvature for Retroreflectors Based on Thermal Reflow

**DOI:** 10.3390/mi15070816

**Published:** 2024-06-25

**Authors:** Yiqiu Yong, Si Chen, Hao Chen, Haixiong Ge, Zongbin Hao

**Affiliations:** 1 Department of Materials Science and Engineering, Jiangsu Key Laboratory of Artificial Functional Materials, College of Engineering and Applied Sciences, Nanjing University, Nanjing 210093, China; mg21340048@smail.nju.edu.cn (Y.Y.); nanocs@smail.nju.edu.cn (S.C.); haixiong@nju.edu.cn (H.G.); 2 National Laboratory of Solid State Microstructures, Collaborative Innovation Center of Advanced Microstructures, Nanjing 210093, China; 602022220005@smail.nju.edu.cn; 3 School of Physics, Nanjing University, Nanjing 210093, China

**Keywords:** microlens array, tunable curvature, high fill factor, microlens-based retroreflector

## Abstract

Retroreflectors are an important optical component, but current retroreflector structures and manufacturing processes are relatively complex. This paper proposes a rapid, low-cost, large-area method for fabricating retroreflectors based on microlens arrays. Tunable microlens arrays with adjustable curvature, fill factor, and sizes were prepared using photolithography and thermal reflow techniques. Subsequently, a two-step nanoimprinting process was used to create a flexible reverse mold and transfer the structure onto the desired substrate. The microlens arrays, with a diameter of 30 μm, a period of 33 μm, a curvature radius ranging from 15.5 to 18.8 μm, and a fill factor ranging from 75.1% to 88.8%, were fabricated this way. In addition, the method also fabricated microlens arrays with diameters ranging from 10 to 80 μm. Retroreflectors were made by sputtering a layer of silver on the MLAs as a reflecting layer, and tests showed that the microlens-based retroreflector exhibited superior retroreflective performance with a wide-angle response of ±75°. Microlens-based retroreflectors have the advantages of simple operation and controllable profiles. The fabrication method in this paper is suitable for large-scale production, providing a new approach to retroreflector design.

## 1. Introduction

A retroreflector is a device that can reflect rays back to the source in almost the same direction with minimal scattering over a wide range of incident angles [1]. Retroreflectors have been widely applied in traffic and safety signs [2,3,4], measurement instruments [5,6,7], optical communication systems [8,9,10,11], automotive lighting [12], and military applications [13,14]. Due to different structures, common retroreflectors include corner cube retroreflectors (CCR, or retroreflecting prisms), hollow retroreflectors, and cat’s eye retroreflectors. CCR and hollow retroreflectors consist of three mutually perpendicular reflecting surfaces, which are simple in structure, but the limit is that their angular responses are only 50.3° and 32.2°, respectively [15]. The cat’s eye retroreflector consists of two concentric hemispheres, which are usually of different diameters. The cat’s-eye retroreflector has better performance than the previous two types of retroreflectors, but the requirement of high-precision alignment of the centers of the two hemispheres makes the preparation process much more complicated. Chan and Ford [16] designed a modulating corner-cube reflector based on one microelectromechanical system (MEMS) mirror that can deform from a flat into a hexagonal array of concave reflective microlenses, which achieved 100 kHz modulation over a 100 nm optical bandwidth and a 35° range of incident angles. Chu et al. [17] prepared a hierarchical photonic structure composed of polystyrene microspheres and a cholesteric assembly of cellulose nanocrystals. This microlens structure can either direct light to be retroreflected on its surface or transmit light to form images. Thus, combining retroreflectors with microlens arrays is a viable approach to broadening the application areas of microlenses.

MLA is one of the key components in optical systems, such as display technology [18,19], integral imaging [20,21,22,23], and optical communication systems [24,25,26]. Three important parameters should be taken into consideration when designing MLAs, including size, curvature, and fill factor (FF). The FF is used to measure the ability of optical components to converge or diverge light, which is defined as the ratio of the effective area to the total area. Increasing the effective area of the microlens array and reducing the gaps between individual microlens units can effectively improve light transmission efficiency. The curvature of the microlenses affects both the focal length and the numerical aperture (NA). The focal length can be calculated using Equation (1), where n is the refractive index of the microlens material.

(1) $$\;f=\frac{h^{2}+r^{2}}{2h\left(n-1\right)}$$

NA determines the resolution and light-gathering ability of the microlens. Higher curvature results in a higher NA, improving resolution while reducing the working distance.

Various methods have been proposed to fabricate MLAs, including ultra-precision mechanical machining, MEMS-based wet etching, wettability-guided dip coating, inkjet printing, the self-assembly method, photoresist thermal reflow, two-photon polymerization, etc. Ultra-precision machining refers to using precision mechanical instruments to manufacture MLAs [27,28,29,30]. This is an effective method for producing large-area MLAs, but due to the single-point process, the operation time and cost will increase. Additionally, the wear and deviation of the cutting tool may lead to a decrease in the uniformity and surface smoothness of the microlenses. MEMS-based wet etching forms concave microlens in an isotropic etch solution, which has smooth surfaces and high uniformity [31,32,33]. However, this method leads to high costs and complicated procedures to generate precise patterns on the mask layer. Inkjet printing, also known as microdroplet jetting, uses a piezo-actuated inkjet nozzle to spray polymer droplets onto the substrate [34,35]. This method has the advantages of simple operation, low cost, and large-scale production. However, because microlenses are fabricated one by one, the position accuracy and uniformity may not be as good as other methods. In addition, the viscosity range of traditional inkjet printing inks is inconsistent with that of typical optical liquids [23]. The typical steps of the self-assembly method [36,37,38,39] involve dropping a solution of particles onto the substrate and allowing them to self-assemble into microlens structures via surface tension and intermolecular forces. This method tends to have more defects over a large area, leading to fluctuations in the accuracy and uniformity of the microlenses. Two-photon polymerization (TPP) [40,41] is a direct laser writing technique that relies on two-photon absorption to induce polymerization in a photosensitive material. TPP offers significant advantages in terms of high resolution and precision. However, its disadvantages, including slow processing speed, high equipment costs, and process complexity, can limit its application in large-scale manufacturing.

Photoresist thermal reflow can fabricate MLAs in large areas with high uniformity and smooth surfaces. Additionally, it does not require complex steps. However, due to objective factors such as photoresists and instruments, as well as subjective factors such as experimental operations, even with the same parameters and preparation process, there may be a little discrepancy in the microlens dimensions of the two samples, reducing the consistency of repeated experiments. Nanoimprint lithography (NIL) can effectively solve this problem because the fabricated flexible mold of MLAs can be repeatedly used multiple times and ensures the high fidelity of the structure. Nanoimprinting is a highly efficient technique for replicating and transferring high-resolution patterns, capable of fabricating structures below 10 nm [42]. Additionally, since nanoimprinting does not require the complex optical systems used in UV lithography, nanoimprinting equipment is more integrated. Compared to lithography machines, nanoimprinting systems significantly reduce both equipment and operational costs [43].

Thus, the combination of thermal reflow technology and NIL technology is a feasible method that combines the advantages of simple and fast operation, good uniformity, and high repeatability. It also allows for large-area fabrication, providing a possible way for mass production.

In this paper, a method combining thermal reflow and NIL technology was proposed to fabricate MLAs on a quartz substrate. MLAs were formed by heating the photoresist pillars slightly above the glass transition temperature, and due to the surface tension, the photoresist flowed to form a smooth hemisphere. Different photomask designs allowed for different sizes of MLA. By adjusting the thickness of the spin-coated photoresist, the curvature of the microlens could be controlled. Chemical vapor deposition of films on the surface of microlenses could effectively increase the fill factor of the microlens array. Using nanoimprints to prepare flexible molds of microlens arrays allowed the structure to be imprinted on different substrates according to requirements. Low surface energy treatment on the microlens surface before nanoimprint facilitated demolding, ensuring the integrity of the structure on the flexible mold and preventing surface contamination of the microlens samples. The reason for not directly fabricating MLAs on a quartz substrate was that the photoresist had a certain light absorption, leading to a negative impact on the optical performance of MLAs. This method not only allowed for the rapid fabrication of microlens arrays but also utilized the fabricated flexible mold to compensate for sample discrepancies introduced during the experimental process, ensuring the consistency of the structure obtained. Furthermore, the MLAs fabricated have high uniformity and smoothness in large areas, making them suitable for mass production.

## 2. Materials and Methods

**Figure 1:**
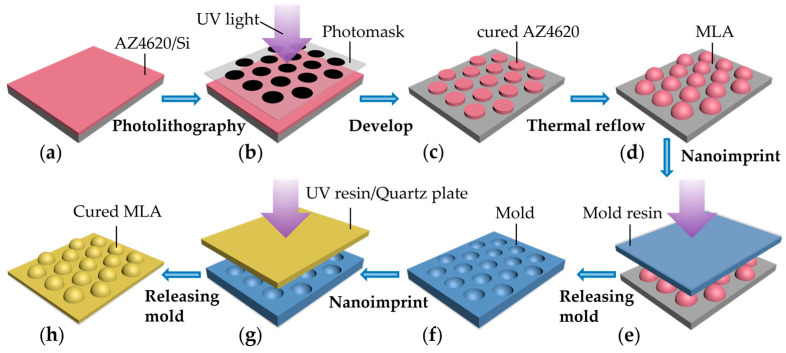
Schematic illustration of the MLA fabrication procedure. (**a**) Spin-coating photoresist AZ4620 on a silicon substrate; (**b**) followed by photolithography; (**c**) cured AZ4620 of the designed pattern; (**d**) MLAs after thermal reflow; (**e**) nanoimprinting; (**f**) obtaining flexible molds of concave structure; (**g**) second step of nanoimprinting; and (**h**) obtaining MLAs on a quartz substrate.

To fabricate MLAs on a transparent quartz substrate, two main steps are required, including (1) the fabrication of MLAs on a silicon substrate and (2) the transfer of the pattern onto a quartz substrate via nanoimprint technology. Figure 1 illustrates the process of fabricating and transferring MLAs. Photoresist AZ4620 (from Merck KGaA, Darmstadt, Germany) was spin-coated onto a silicon substrate (Figure 1a) and prebaked for 3 min at 100 °C. After cooling for over half an hour, the substrate with photoresist was exposed to UV light in the lithography machine (Karl Suss MA6, from SUSS MicroTec, Garching, Germany ). If not sufficiently cooled before the next step, it will lead to contamination of the photomask with uncured photoresist under the hard-contact lithography mode. The photomask was patterned with a hexagon-arranged array of opaque spots (Figure 1b). The substrate was immersed in 2.38% TMAH (Tetramethylammonium Hydroxide, from Merck KGaA) to remove the uncured photoresist (Figure 1c). Then, the substrate was placed on a hotplate for thermal reflow at 160 °C for 40 min, forming microlenses with smooth surfaces. After cooling down, a solid microlens array was obtained due to surface tension (Figure 1d).

Transferring the pattern from the silicon substrate to the quartz substrate required two nanoimprint steps. Before nanoimprinting, a low-surface-energy treatment was applied on the MLA surface by using 1H, 1H, 2H, and 2H-perfluoroecyltrichlorosilane (FDTS), which facilitated demolding in the following step. Then, a mold resin (from PRINANO Technology Co. Ltd., Hangzhou, China) was spin-coated on the MLA and covered with a flexible poly(ethylene terephthalate) (PET) (Figure 1e). After nanoimprinting and curing by UV light, a flexible mold of microlens was prepared (Figure 1f). An adhesive resin and UV-resin (from PRINANO Technology Co. Ltd.) were spin-coated on a quartz substrate, respectively, followed by covering the flexible mold onto it, imprinting, and being exposed to UV light (Figure 1g). Crucially, the role of the adhesive resin was to enhance the adhesion between UV-resin and substrate. Finally, the mold was released, and the MLAs were successfully transferred onto a transparent quartz substrate (Figure 1h). Combining thermal reflow and NIL technology, this method allows for the rapid and simple fabrication of large-area MLAs, demonstrating smooth and uniform structures with high consistency even at a large scale.

## 3. Results and Discussion

The size, curvature, and fill factor (FF) of microlens are important factors affecting imaging performance. This study employed a direct method to control the parameters of microlens arrays and fabricated an approximation of an ideal hemispherical shape with high FF. Two factors should be taken into consideration when choosing a photoresist: an appropriate thickness matching the structure size and a suitable glass transition temperature. AZ4620 meets the above requirements with a thickness of 7.5 μm at 4000 rpm and a glass transition temperature of about 125 °C. The relationship between the photoresist thickness, the spin-coating speed, and the photoresist concentration is given by Equation (2):

(2) $$\;h=\frac{kc}{\sqrt{\omega }}$$

where h is the thickness, k is a constant related to the resin, c is the concentration of the resin, and *ω* is the spin-coating speed.

**Figure 2:**
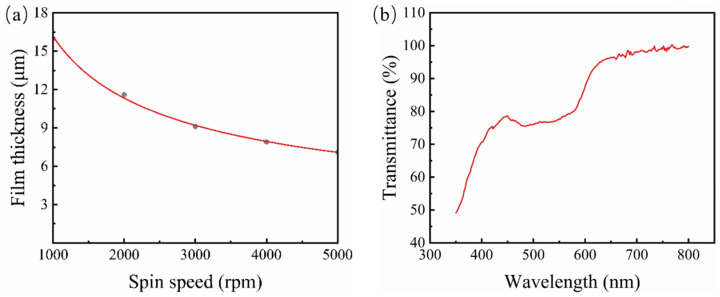
(**a**) The curve of the relationship between the film thickness and spin speed of photoresist AZ4620. (**b**) Optical transmittance image of AZ4620 photoresist film.

**Figure 3:**
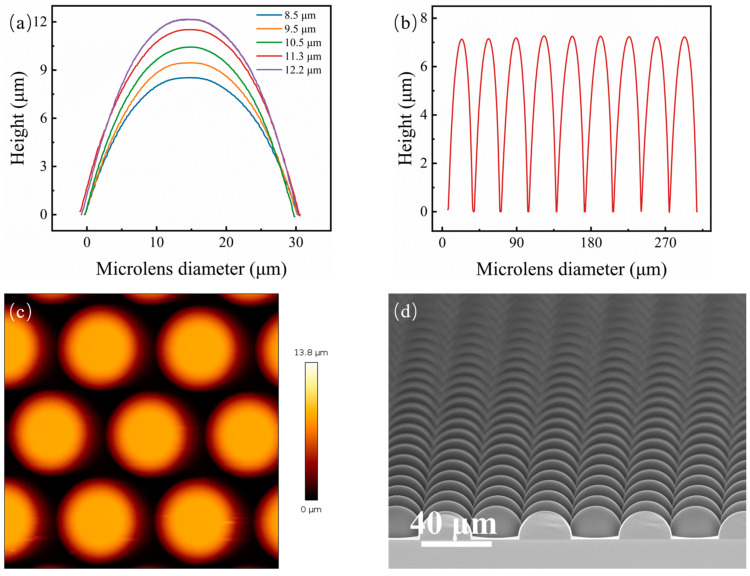
(**a**) Cross-section profiles of the MLA with different curvatures; (**b**) cross-section profiles of the MLA. (**c**) AFM scans were taken over a 100 × 100 μm^2^ area of the MLAs. (**d**) Microlenses close to hemispherical shape.

Spin-coating photoresists with different speeds resulted in different photoresist thicknesses; thus, substrates with varying photoresist pillar heights were obtained, consequently controlling the height of the microlens after thermal reflow as well as curvature. The curve showing the relationship between the thickness of AZ4620 and the spin coating speed is shown in Figure 2a. Figure 2b shows the transmission spectrum of the AZ4620 photoresist film when spin-coated at a rate of 4000 rpm. It can be seen that the photoresist has significant light absorption in the visible range, especially below 600 nm. Directly fabricating the photoresist microlens array on a quartz substrate would affect the optical performance. Therefore, this paper adopted a two-step nanoimprinting method for pattern transfer. Figure 3a,b shows the cross-sectional profile images of MLAs observed by the KLA Profilometer, and therefore, it can be inferred that MLAs fabricated in this method had different curvatures and uniform profiles. The surface roughness of the microlens was measured by atomic force microscopy (AFM). As shown in Figure 3c, in a scanning area of 100 × 100 μm^2^, the roughness of the obtained MLAs was 3.52 nm RMS. Therefore, it can be seen that the thermal reflow method can be used to fabricate uniform and smooth microlens arrays, which are crucial for improving optical performance. Obviously, with the increase in photoresist thickness, the curvature of the microlens increased. This method successfully fabricated microlenses close to a hemispherical shape when the spin-coating speed was 1000 rpm (Figure 3d).

**Figure 4:**
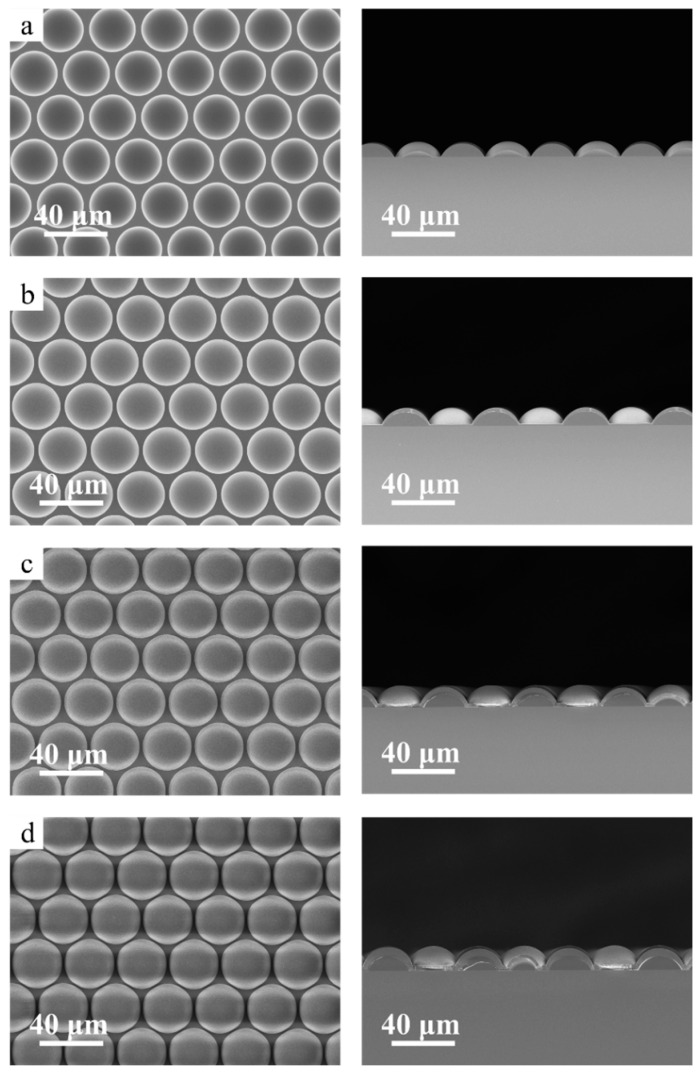
Top and their corresponding cross-sectional profile SEM images of SiO_2_ deposition on MLAs, with a deposition time of (**a**) 0 min, (**b**) 45 min, (**c**) 90 min, and (**d**) 135 min.

**Figure 5:**
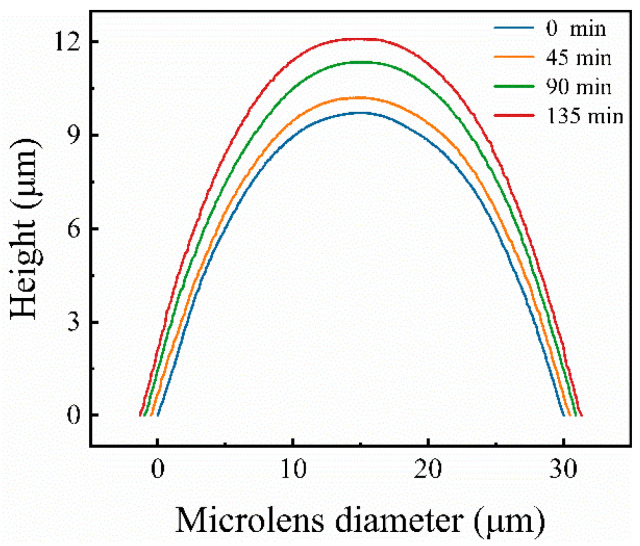
Cross-section profile of the MLAs that were deposited with SiO_2_.

This study used chemical vapor deposition to increase the FF of the microlenses. SiO_2_ was deposited on the surface of the microlens using PECVD (Plasmalab System 80Plus, from Oxford Instruments, Abingdon, United Kingdom). Two important factors need to be considered when it comes to deposition time: the deposition rate and the desired thickness. The deposition rate of the PECVD device we used was approximately 30 nm/min. However, this rate can be greatly influenced by factors such as the deposition thickness, size, and shape of the structures. To enhance the filling factor as much as possible, the desired thickness is 1.5 μm, half of the gap width between two microlens cells. However, the deposition rate on the sidewalls is typically lower than on the top surfaces because of directional plasma flux, limited diffusion to the sidewalls, the shadowing effect, etc. Thus, the increase in film thickness at the side walls is less than at the top surface. Deposition times of 45, 90, and 135 min were chosen to show a significant increase in the filling factor. Further extension of the deposition time did not contribute much to the fill factor but significantly increased the cost or might even begin to degrade optical performance due to excessive material build-up. By changing the deposition time of the film to 45 min, 90 min, and 135 min, the FF of the microlenses could be effectively adjusted from 75.1% to 88.8%. The cross-section of the deposited microlenses was characterized in Figure 4 using SEM and compared with microlenses without film deposition. Figure 5 shows the cross-sectional profile images of MLAs obtained by the KLA Profilometer. Thus, the FF was effectively improved in this way.

**Figure 6:**
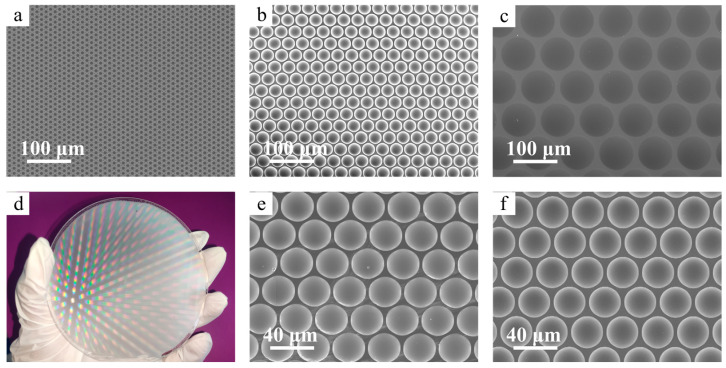
SEM images of MLAs with a diameter of (**a**) 10 μm, (**b**) 30 μm, and (**c**) 80 μm. (**d**) MLAs with a diameter of 10 μm nanoimprinted on a quartz substrate. SEM images of (**e**) a flexible reverse mold and (**f**) MLA on a quartz substrate.

In addition to controlling the curvature and FF of the microlens, this method can also fabricate microlens of different sizes to meet the requirements of various optical applications. In this work, the designed diameters of the spots arrays of three photomasks were 10 μm, 30 μm, and 80 μm, respectively, while the center distances were 12 μm, 33 μm, and 85 μm, respectively. SEM images of different sizes of microlens arrays prepared in this way are shown in Figure 6. It can be observed that the diameters of microlenses with different sizes all exhibited consistent patterns on the masks. The structures of the microlens arrays showed high uniformity and high consistency with the designed patterns. This demonstrates that this method can control the curvature and size of the microlens, allowing for the design and fabrication of microlens with different focal lengths and sizes according to optical requirements. Another key factor is the area of the samples that can be fabricated. Therefore, attempts were made, and microlens arrays on a four-inch silicon wafer were successfully fabricated, which were then transferred onto a quartz substrate by nanoimprinting, as shown in Figure 6d. By two-step nanoimprinting, a flexible reverse mold of MLA on PET was prepared, and the structure of the flexible mold was accurately transferred onto a quartz substrate. SEM images of MLA on the flexible mold and quartz substrate are presented in Figure 6d,e, which indicate that the two-step nanoimprinting ensured the faithful replication of the structure onto the desired substrate with few defects. These results suggest that the fabrication of large-area, uniform, and controllable MLAs through UV lithography, thermal reflow, and nanoimprinting is feasible and easily operated, having the potential for industrial-scale production.

**Figure 7:**
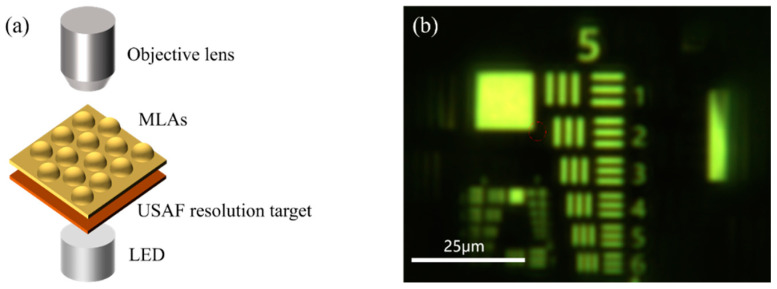
(**a**) Schematic diagram of the USAF standard resolution test setup. (**b**) Resolution target observed through the MLA.

MLAs with a diameter of 80 μm were nanoimprinted onto a quartz substrate, and a measurement of resolution was made by observing the United States Air Force (USAF) resolution target. The optical measurement setup is shown in Figure 7a. The result shown in Figure 7b indicates that element 6 of group 5 on the resolution target can be distinguished at most, which means the resolution of the MLA is approximately 57 lp/mm. This indicates that the fabricated MLAs have excellent imaging performance, further demonstrating their smooth surface morphology and high fidelity of nanoimprinting.

**Figure 8:**
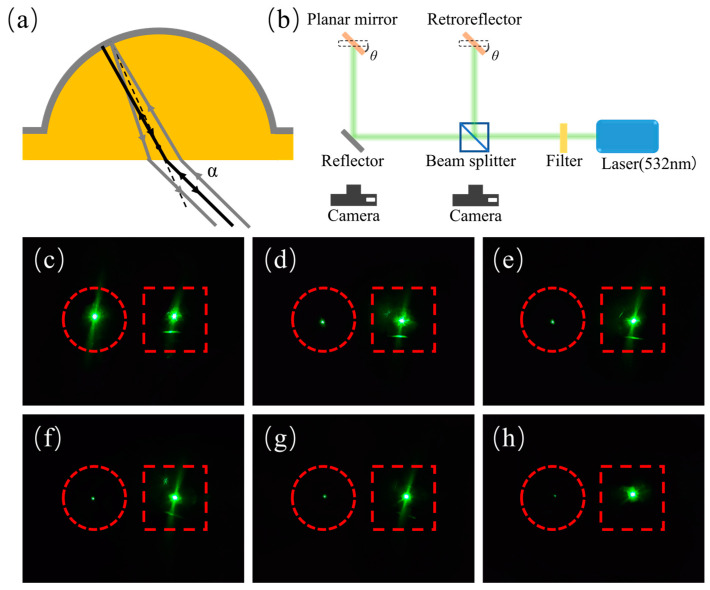
(**a**) Optical path through the retroreflector. (**b**) Schematic diagram of the setup for testing the retroreflective performance of fabricated retroreflectors. The reflected lights of the planar mirror and retroreflector R3 were observed from the direction of the incident light, which was at an angle of (**c**) 0°, (**d**) 15°, (**e**) 30°, (**f**) 45°, (**g**) 60°, and (**h**) 75°.

Patterns of the three samples with different curvatures and FF were transferred to quartz substrates, respectively. To obtain a retroreflector based on MLAs, a layer of 100 nm Ag was sputtered by a magnetron sputtering coating machine (Leica EM ACE600, from Leica Microsystems, Wetzlar, Germany) onto the MLAs on a quartz substrate. The three retroreflectors are named R1, R2, and R3, respectively, and the parameters of the three samples are listed in Table 1. The optical path schematic diagram of the retroreflector is shown in Figure 8a. According to the imaging principle of the concave mirror, the parallel incident light passing through or close to the spherical center of a concave mirror, which is called paraxial light, is retroreflected back along the incident light direction.

**Figure 9:**
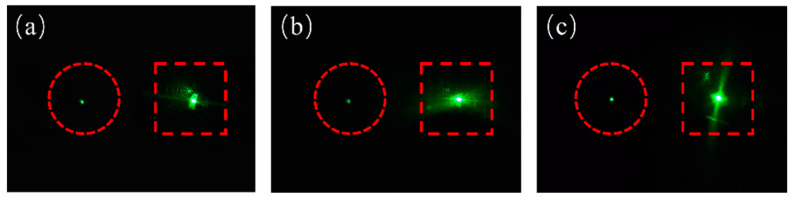
The reflected lights of the planar mirror and retroreflector were observed from the direction of the incident light, which was at an angle of 45° of (**a**) R1, (**b**) R2, and (**c**) R3.

The retroreflective performance was tested using the setup as shown in the schematic diagram in Figure 8b. In this setup, a beam of 532 nm green light was emitted from the laser source, passed through a filter, and split into two identical beams by a beam splitter. The two beams of light were incident at 0°, 15°, 30°, 45°, 60°, and 75° angles on a planar Ag mirror without MLA structure and R3, respectively. Observing the reflected light from the direction of the incident light, as shown in Figure 8c–h, it can be seen that when the incident light was perpendicular to the samples, the reflected lights of the planar mirror and the retroreflector had similar brightness. However, when the angle of the incident light deviated, the reflected light from the planar mirror noticeably weakened. This is because specular reflection reflects most of the light on the other side of the normal. In contrast, the retroreflector can still observe strong reflected light. Additionally, as the angle of incident light increased, the reflected light observable in front of the planar mirror became weaker, while the retroreflector’s reflected light did not show significant attenuation. It means the efficient reflected angle of R3 can reach ±75°. Meanwhile, retroreflective performance tests were conducted on three MLA samples with different curvatures and FF, and the results are shown in Figure 9. The incident angle was set to 45°, and it can be observed that all three samples exhibited bright retroreflected light, indicating good retroreflective performance. In addition, another important parameter is the divergence angle of the reflected beam. It is the angle at which a light beam spreads out as it propagates. Specifically, it is the angle between the central axis of the light beam and the edge of the beam after it has traveled a certain distance. The divergence angle is commonly used to describe the spreading of light beams. The divergence angle of the reflected beam we measured was about 1.7°, which means that from a long distance, strong retroreflected light can be observed.

Therefore, it can be seen that retroreflectors based on microlens arrays exhibit excellent retroreflective performance. For retroreflectors, changes in curvature have a negligible impact on their performance. However, in other application fields, such as integral imaging, curvature has an important influence on optical performance. Combining the advantages of large-area fabrication and imprinting of microlens arrays in this method, it provides a feasible approach for the application of retroreflectors based on microlens arrays.

## 4. Conclusions

In this work, the proposed method based on thermal reflow, coupled with NIL technology, presents a viable approach for the fabrication of large-area microlens arrays. By changing the thickness of the photoresist, the curvature radius of microlenses can be controlled from 15.5 to 18.8 μm. By depositing SiO_2_, the FF was improved from 75.1% to 88.8%. By designing photomasks of different sizes, the diameter of the microlens can be altered from 10 to 80 μm. An area of 4 inch microlens arrays has been successfully fabricated, which was accurately imprinted on a quartz substrate. A layer of Ag was sputtered on the MLAs, and thus, the obtained retroreflectors were tested for their retroreflective performance. Observing the retroreflected light from the direction of the incident light, the retroreflectors exhibited significantly better reflected light intensity than a planar mirror at an incident angle range of ±75°. The microlens-based retroreflector provides a new and simple way to design and fabricate retroreflectors with high retroreflective performance and a large area.

## Figures and Tables

**Table 1:** Parameters of the MLAs fabricated into retroreflectors.

	Microlens Height (μm)	Diameter (μm)	Curvature Radius (μm)	Fill Factor
R1	8.1	30.9	18.8	79.3%
R2	10.5	31.6	17.1	82.9%
R3	15.7	31.7	15.9	83.7%

## Data Availability

Data are contained within the article.
